# Measuring ensemble interdependence in a string quartet through analysis of multidimensional performance data

**DOI:** 10.3389/fpsyg.2014.00963

**Published:** 2014-09-02

**Authors:** Panos Papiotis, Marco Marchini, Alfonso Perez-Carrillo, Esteban Maestre

**Affiliations:** ^1^Music Technology Group, Department of Information and Communication Technologies, Universitat Pompeu FabraBarcelona, Spain; ^2^Input Devices and Music Interaction Laboratory, Centre for Interdisciplinary Research in Music Media and Technology, Schulich School of Music, McGill UniversityMontreal, QC, Canada; ^3^Computational Acoustic Modeling Laboratory, Centre for Interdisciplinary Research in Music Media and Technology, Schulich School of Music, McGill UniversityMontreal, QC, Canada

**Keywords:** interdependence, string quartet, ensemble performance, signal processing, motion capture, mutual information, nonlinear coupling, granger causality

## Abstract

In a musical ensemble such as a string quartet, the musicians interact and influence each other's actions in several aspects of the performance simultaneously in order to achieve a common aesthetic goal. In this article, we present and evaluate a computational approach for measuring the degree to which these interactions exist in a given performance. We recorded a number of string quartet exercises under two experimental conditions (*solo* and *ensemble*), acquiring both audio and bowing motion data. Numerical features in the form of time series were extracted from the data as performance descriptors representative of four distinct dimensions of the performance: Intonation, Dynamics, Timbre, and Tempo. Four different interdependence estimation methods (two linear and two nonlinear) were applied to the extracted features in order to assess the overall level of interdependence between the four musicians. The obtained results suggest that it is possible to correctly discriminate between the two experimental conditions by quantifying interdependence between the musicians in each of the studied performance dimensions; the nonlinear methods appear to perform best for most of the numerical features tested. Moreover, by using the *solo* recordings as a reference to which the *ensemble* recordings are contrasted, it is feasible to compare the amount of interdependence that is established between the musicians in a given performance dimension across all exercises, and relate the results to the underlying goal of the exercise. We discuss our findings in the context of ensemble performance research, the current limitations of our approach, and the ways in which it can be expanded and consolidated.

## Introduction

Music performance is, as one of the major performing arts, a social experience. The performer, the audience as well as the composer interact with each other in both direct and indirect ways; which makes music performance a fertile research topic when studying human interaction. In this article, we place the focus on the interaction between the members of a musical ensemble and the interdependence that is established between them as a result.

Musicians playing together must not only synchronize their internal timekeeping mechanisms, but also jointly shape the more nuanced aspects of their performance such as intonation or dynamics in order to achieve a (presumably) shared aesthetic goal (Keller, 2008). In order to achieve this while performing, the members of the ensemble communicate nonverbally with each other to exchange information using auditory as well as visual feedback (Goebl and Palmer, 2009; D'Ausilio et al., 2012).

The mechanisms that enable social interaction between the performers also depend on the type of ensemble. Larger ensembles are usually led by a conductor who acts as a leader in terms of temporal coordination (Rasch, 1988; Luck and Toiviainen, 2006), evaluates the performance and provides additional instructions on how it should be carried out. On the other hand, small ensembles are typically conductor-less, and such tasks are placed upon the musicians themselves. In small ensembles, each performer usually has their individual part, while in large ensembles it is common for given sub-sections of the same instrument family to have identical parts—examples of this are first and second violin sections within a symphonic orchestra. The subject of research in this study is the string quartet, a small conductor-less ensemble of four musicians (two violins, a viola and a cello) where every musician has their own individual part. Although the first violinist is traditionally considered the “leader” of a string quartet, this cannot be always expected to be the case; moreover, there is no guarantee that he/she will have the greatest decision-making influence in the group (Young and Colman, 1979).

In small conductor-less ensembles such as a string quartet, each musician must divide their attention between shaping their own performance and aligning their expressive choices with the rest of the ensemble. Such cognitive mechanisms, along with anticipatory and timekeeping mechanisms, are discussed at length in Keller's work (Keller, 2008; Pecenka and Keller, 2009; Keller and Appel, 2010). Timekeeping and synchronization mechanisms are also extensively investigated in the so-called *tapping literature* (a literature review of which can be found in Repp, 2005), a combination of theoretical and empirical research on sensorimotor synchronization based on experiments featuring rhythmic tapping tasks. Among the many findings, there are two important synchronization phenomena of special interest for the scope of this study: *phase correction* and *period correction*; viewing tempo as an oscillating system, these two mechanisms account for short-term and long-term adjustment mechanisms, respectively.

Performance in an ensemble typically involves more complex temporal interactions than the ones observed in a rhythmic tapping task. Synchronization in more natural performance conditions has been investigated by Goebl and Palmer (2009), who demonstrated the importance of both auditory and visual feedback in piano duet performances as well as how the absence of one type of feedback affects the musicians' reliance on the other. This study also used solo performances as a baseline measure of timing similarity, a technique that is adopted in our study. Besides controlling the existence and type of feedback, the authors also assigned musical roles (leader/follower) to the musicians. While some effects of the musical role on the performance could be observed, the results combined with the participants' comments suggest that cooperation is preferred to strict musical roles. Empirical work done by Moore and Chen (2010) on string quartet performance also seems to support this conclusion; despite observing high temporal precision across the entire quartet, they did not encounter evidence for one individual performer being responsible for the achievement of synchrony. In their words: “In the complex performance studied here every response is conceivably a stimulus, every stimulus conceivably a response; but neither can be identified” (pp. 413, section 4.3 “Timing and interactions”).

The lack of explicitly assigned roles in an ensemble's performance does not necessarily imply a lack of increased influence for specific members of the ensemble. In a comprehensive data-driven study carried out by Wing et al. (2014), two different string quartets were asked to perform a short excerpt while introducing unrehearsed expressive deviations in terms of timing in their performance. The authors utilized phase correction measures to quantify the amount of effort employed by one performer to adjust to the tempo of others, showing different results for each quartet. In one quartet, the first violinist exhibited less adjustment to the others than vice versa, while in the second quartet, the levels of correction by the first violinist matched those exhibited by the others; a result that highlights how quartets can employ different strategies ranging from an “autocracy” to a “democracy” when it comes to tempo coordination. Another work that deals with social interaction in string quartet performance has been carried out by Glowinski et al. (2013). Focusing on body movement, its relation to expressivity, and the purpose it serves in coordinated action, they highlight the challenges of carrying out empirical research on realistically complex scenarios.

When it comes to other performance dimensions such as intonation, dynamics, or timbre, there is little work to be found; the large majority of the literature is focused on temporal synchronization. One of the few related studies deals with singing in barbershop quartets (Kalin, 2005), where the results strongly suggested that the singers strive to separate their formants from one another in order to facilitate correct intonation; which also suggests that modulations in one dimension of the performance (in this case timbre) might be employed to achieve optimal coordination in another (intonation). A methodology for analyzing polyphonic vocal performances has also been outlined by Devaney and Ellis (2008). While differences between solo and ensemble performance have been observed both for dynamics (Goodman, 2002) and intonation (Nickerson, 1949; Mason, 1960), it is still unclear what their function is within the context of musical interaction. Recent work on how intonation and dynamics adjustments contain information on the coordination between musicians has been presented in Papiotis et al. (2012).

So far, the literature mostly addresses the question of *how* ensemble musicians coordinate their actions to bring about the optimal co-variation of expressive performance parameters. There are however several factors that can contribute to, or hinder coordinated action: the technical difficulty of the piece in relation to the capabilities of the performers, the conditions under which the performance takes place, the performers' familiarity with the piece and with each other and the performers' condition during the performance, to name a few. Besides practical factors, joint musical action assumes that a common goal is shared by the musicians—an assumption that cannot be always considered fully true in every ensemble's performance. In this article, we would like to pose a question different than *how* musicians interact: given a certain performance by an ensemble, is it possible to measure *whether* the musicians are indeed interacting and adapting to each other to achieve a coordinated result? This question is especially relevant when studying performance dimensions such as Intonation or Dynamics, rather than synchronization—since the establishment of a common tempo is the first fundamental step in order to achieve joint musical action.

We therefore wish to test the following hypothesis: for each dimension of the performance individually, it is possible to measure whether the musicians are indeed interacting with each other by measuring the interdependence between time series features extracted from the acoustic result of the performance. In other words, to investigate whether the variations in the way the musicians produce sound contain information about the interaction between them that is observable using current computational methods of interdependence. An extension to our hypothesis is that it is not only possible to measure *whether* the performers are interacting or not, but to also compare *how much* the degree of interaction in a given performance dimension varies across different pieces/exercises. In this sense, it is not our main goal to directly contribute on understanding how musical interaction is achieved, but rather propose and evaluate a new set of tools that can be subsequently used for contributing to future research on musical interaction.

To this end, we have carried out a set of experiments with a professional string quartet performing under two different experimental conditions; *solo* and *ensemble*. A set of performance descriptors in the form of time series is extracted from each musician's individual sound and sound-producing gestures, each related to a single dimension of the performance (intonation, dynamics, timbre, and timing). We quantify the amount of interdependence between these descriptors across the four musicians for each of the dimensions using a set of computational methods originating from different academic disciplines. A statistical test is performed on the obtained results in order to assess whether significantly higher interdependence is encountered for the *ensemble* condition in comparison to the *solo* condition. Finally, we calculate the variation of interdependence estimations across the different exercises and relate the results to the underlying musical scores.

## Materials and data processing

This section outlines the experiment (in terms of materials and design), the subjects, and conditions under which it was carried out, the data acquisition methodology, and the steps for data pre-processing and feature extraction.

### Experiment design

Two main considerations were taken into account during the design of the experiment. The first one was ensuring that each chosen exercise has a simple shared goal that is clearly understood by the performers; the sharing of a common interpretation is identified as an instrumental aspect of ensemble performance (Keller, 2008). Our selection of the musical scores given to the performers was therefore focused on this.

The second consideration was having a set of *ground truth* data through which comparisons could be as unambiguous as possible. It was essential that the conditions under which the data was gathered reflect the studied phenomenon; and such data can only originate from an experiment whose design is focused around having a clear condition where *zero* interdependence among the musicians exists, and a case where *some* interdependence exists. The chosen *solo* and *ensemble* experimental conditions reflect our attempt at achieving such distinct examples.

#### Selection of scores

The scores were selected from an exercise handbook for string quartets (Heimann, 2007), specifically designed to assist in strengthening the interdependence among the members of the ensemble. These exercises consist of short, simple musical tasks whose difficulty lies in achieving overall cooperation rather than correctly performing one's individual task successfully. Six exercises were chosen from the handbook; with each exercise focusing on a single dimension of the performance. Table 1 contains a summary of the exercises along with a short description for each one.

**Table 1:** **Description of the recorded exercises along with their durations (minutes:seconds)**.

**ID**	**Dimension**	**Description**	**Score duration**	**Ensemble duration**	**Solo duration (average and sdev)**
I1	Intonation	“Vertical listening,” the ability to adjust one's intonation according to the intonation of the rest of the ensemble	2:12	2:24	μ = 2:12 σ = 0:09
D1	Dynamics	Immediate (“subito”) changes in dynamics	0:56	1:03	μ = 0:57 σ = 0:04
D2	Dynamics	Gradual (“crescendo/decrescendo”) changes in dynamics	0:48	0:52	μ = 0:47 σ = 0:05
R1	Rhythm	Small changes in tempo (“poco piu/meno mosso”)	1:20	1:00	μ = 0:57 σ = 0:06
R2	Rhythm	Different degrees of rhythmic syncopation	1:20	1:14	μ = 1:19 σ = 0:11
T1	Timbre	Similar tone quality for different bow-string contact points (“sul tasto/sul ponticello”) and different dynamics levels	0:56	1:05	μ = 0:56 σ = 0:04

**Figure 1:**
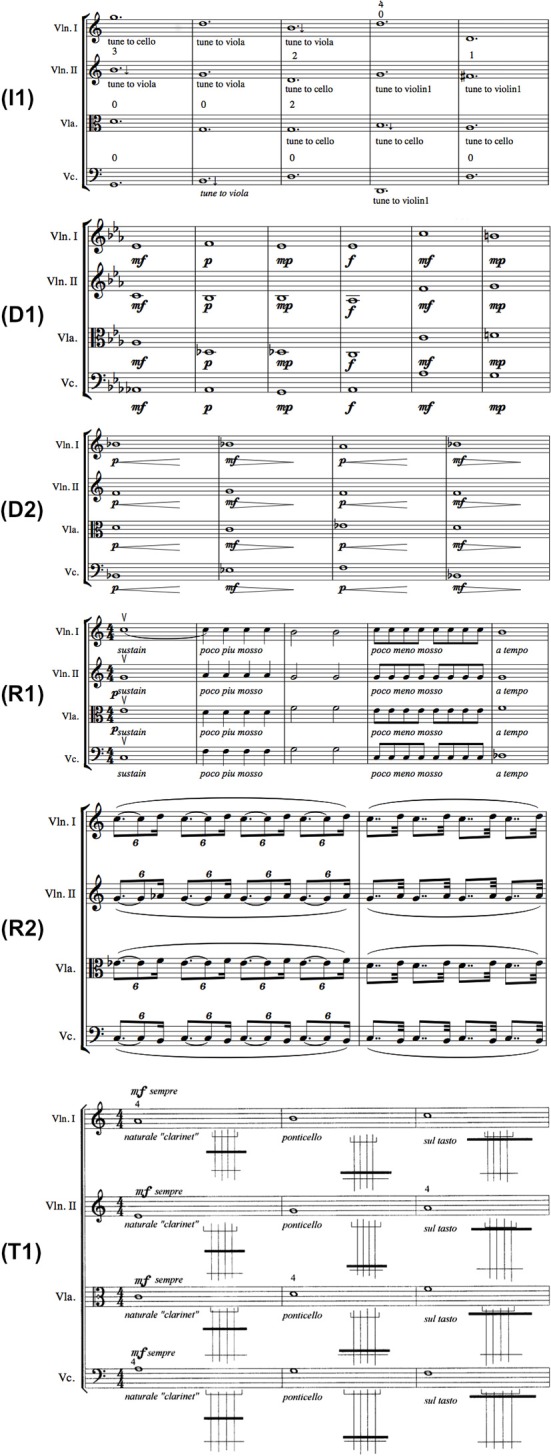
**Score excerpts from all exercises used in the experiment**. (Legend: I1, Intonation exercise; D1, Dynamics exercise nr.1; D2, Dynamics exercise nr.2; R1, Rhythm exercise nr.1; R2, Rhythm exercise nr.2; T1, Timbre exercise).

Each exercise was accompanied by a set of instructions from the composer that were presented as the shared goal of the exercise; examples include “the quartet should sound as one instrument” or “the crescendi and decrescendi must start at the same time and end at the same time.” Besides textual instructions, the intonation and timbre exercises also featured visual annotations that aided in comprehending the goal of the exercise, with the intonation exercise featuring an upward/downward arrow denoting a departure from equal temperament to just intonation, and the timbre exercise featuring a small visual aid denoting the distance of the bow from the instrument bridge. A short excerpt of each exercise can be seen in Figure 1. In all exercises, the musicians' parts are homophonic and generally contain isochronous rhythms.

#### Subjects and conditions

The subjects were members of a professional string quartet that had already been performing together for more than a year, including public performances. The mean age of the musicians was 30 years old (σ = 2.9), and all of them had been practicing their respective instruments for at least 20 years, including at least 10 years of professional experience as members of musical ensembles (chamber orchestras, symphonic orchestras, string quartets). They were all compensated for their participation in the experiment, and signed an agreement (jointly prepared by the participating academic institutions) regarding the use of their data and experimental conditions.

The experiment was conducted in two main experimental conditions, *solo* and *ensemble*. In the *solo* condition, each musician performed their part alone using a stripped-down version of the score; meaning they had no access to the other musicians' scores nor the textual instructions (or annotations for the case of intonation) therein. Essentially, the *solo* condition represents a case where both the shared goal and the communication channels between the musicians are absent. Each musician was provided with their solo version of the score prior to the actual recording, and was left to rehearse their part alone for approximately 15 min per exercise. We provided the musicians with four bars of a metronome click immediately before recording as an indication of the desired tempo.

Following the *solo* condition, the musicians were given the complete quartet's score along with the composer's instructions. They were then left to rehearse the exercise for a short period (10–15 min) or until they felt they were achieving the exercise's shared goal satisfyingly. We did not interfere with the musicians during this rehearsal period. While the rehearsal condition was also recorded, it was not carried out under controlled conditions and the performance was frequently interrupted by the quartet to discuss a particular passage or take notes. For that reason, we decided to exclude it from this study; the importance and role of the rehearsal process in string quartets has also been highlighted in other work (Davidson and King, 2004).

Finally, following rehearsal, the musicians were recorded in the *ensemble* condition; similar to the *solo* condition, they were provided with four bars of a metronome click immediately before recording. For the case of the intonation exercise, we carried out two separate *ensemble* recordings: one without the annotations provided by the composer, and one with the annotations. This was made to ensure that the *solo* version of the intonation exercise was not too far removed from the *ensemble* version. For the case of the timbre exercise, the provided annotations were present in both the *solo* and *ensemble* scores since they referred to the playing technique rather than the common goal of the ensemble.

The experiment was carried out over the course of several days; all of the *solo* condition recordings were carried out during the first day, with the rehearsals and *ensemble* condition recordings spread out throughout the rest of the days. The ordering of the recording sessions was the following: *solo* violin 1 (all exercises), *solo* violin 2 (all exercises), *solo* viola (all exercises), *solo* cello (all exercises), *ensemble* dynamics (D1), *ensemble* dynamics (D2), *ensemble* intonation (I1), *ensemble* rhythm (R1), *ensemble* rhythm (R2), *ensemble* timbre (T1). The consistent *solo-ensemble* ordering was a necessity of the experimental design, as the lack of familiarity with the scores of the rest of the ensemble is a core characteristic of the *solo* condition. While this could potentially entail a risk of order effects, the simplicity of the musicians' parts in each exercise reduces the possibility of becoming more familiar through additional exposure.

### Data acquisition

Individual audio from each performer was acquired through the use of piezoelectric pickups attached to the bridge of instruments; the recorded audio was sampled at 44,100 Hz. Pickup gains were manually adjusted in the mixing console with the aid of level meters in the recording equipment to avoid the clipping of individual audios and to ensure equivalent levels of intensity led to equivalent signal amplitudes. We also simultaneously acquired bowing motion data at a sample rate of 240 Hz by means of a Polhemus Liberty wired motion capture system as detailed in Maestre (2009). In a post-acquisition step, the synchronization of audio and motion capture data was reviewed through the alignment of linear timecode timestamps.

### Data processing

#### Score-performance alignment

The existence of an underlying musical score for each of the recordings provides a reference to which each performance can be compared. In order to obtain this absolute reference, we performed a precise score-performance alignment for each recording, obtaining note onset and note offset times expressed in milliseconds. This procedure was carried out by means of a semi-automatic score-performance alignment algorithm which utilizes both the audio as well as motion data to iteratively estimate note boundaries (Maestre, 2009). Estimated note onsets and offsets were then manually inspected to correct possible segmentation errors.

#### Feature extraction

The next step to our data processing pipeline is to extract numerical features in the form of time series from our data which characterize the performance in terms of four dimensions: Intonation, Dynamics, Tempo, and Timbre.

*Intonation* refers to the accuracy of the produced pitch for a musical performance. String instruments are fretless and are therefore capable of producing continuous pitch; the musicians must constantly perform adjustments to their intonation in order to achieve harmonic consonance for the overall sound. When each performer has a different score, the achievement of consonance is further complicated by the choice of *tuning temperament*—fine adjustments to the note intervals that can vary from a twelve-tone equal temperament (i.e., each pair of subsequent notes having an identical frequency ratio) to just intonation (intervals defined by the harmonic series), among several other temperament systems.

We remind the reader that our goal is not to observe whether the performers are indeed achieving consonance or not, but to measure whether the adjustments to their intonation are influenced by the rest of the ensemble. Given that each performer has their own part, a direct comparison of each performer's produced pitch is not possible; an objective reference is needed in order to express intonation adjustments in a common scale across performers and different melodic lines. We utilized the score as “reference pitch,” i.e., perfect non-adjusted intonation (according to the equal temperament tuning system), from which the performer's deviations can be calculated.

Our pitch deviation feature is therefore defined as follows. For every recorded performance, the fundamental frequency of the audio signal is estimated using the YIN algorithm (De Cheveigné and Kawahara, 2002). YIN's output is pitch expressed in a linear scale of octaves (with 0 being 440 Hz, 1 being 880 Hz etc.). For a given recording *r*_1_, let us define *f*^*est(r*_1_)^ as the estimated pitch for that recording and *f*^*score(r*_1_)^ as the reference pitch based on the score. By computing the difference between the two, we can obtain the pitch deviation δ*f*^*r*1^:

(1) $$\delta f^{r1}=f^{est(r_{1})}-f^{score(r_{1})}$$

**Figure 2:**
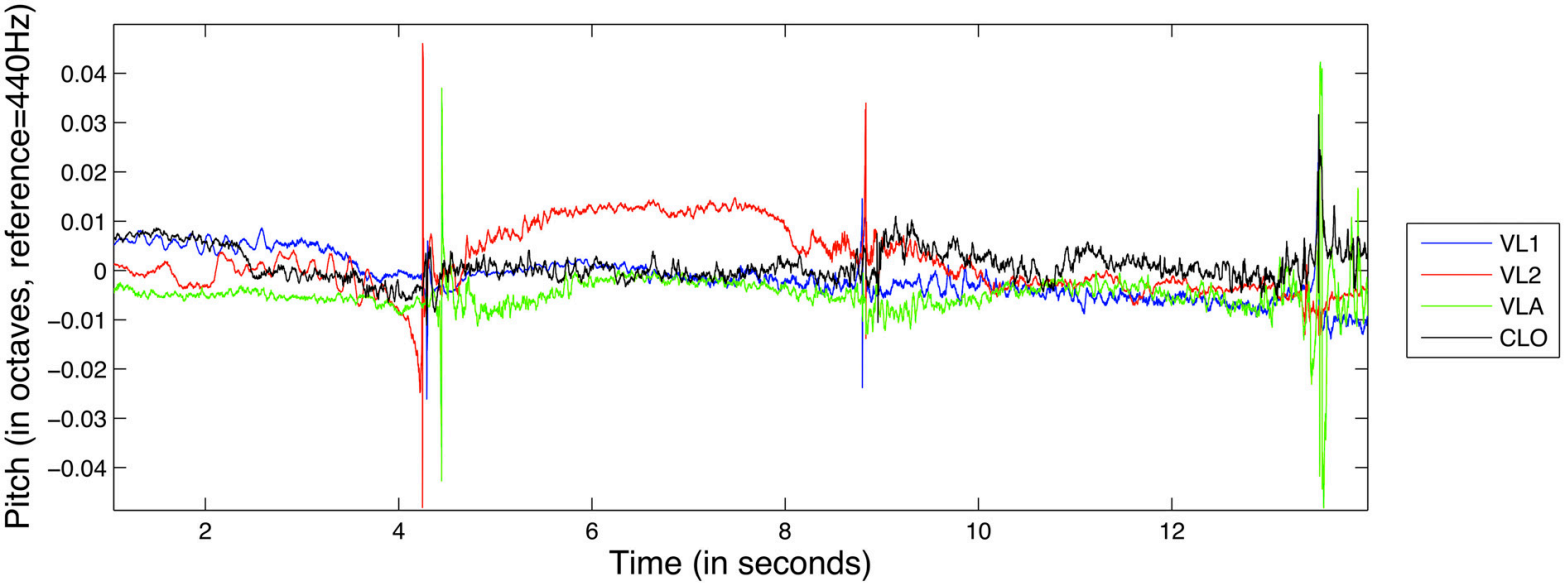
**Excerpt from the pitch deviation feature from exercise D1, for all instruments**.

An example of the pitch deviation feature for the D1 exercise can be seen in Figure 2.

*Dynamics* refer to the varying levels of loudness or sound intensity in a musical performance. For every recording, we estimate loudness (in dB) as follows. The recorded audio signal is divided into frames of 33 ms with a 97.5% overlap. Let (*x*^*i*^_1_,…,*x^i^_n_*) be the samples of the *i*_th_ frame: for each frame we compute the Root Mean Square (RMS) of its samples:

(2) $$RMS\left(i\right)=\;\frac{1}{n}\sqrt{{\left(x_{1}^{i}\right)}^{2}+\cdots +{\left(x_{n}^{i}\right)}^{2}}$$

Finally, we express the *RMS* value in dB:

(3) $$logRMS\left(i\right)=20\log_{10}\left(RMS(i)\right)$$

We perform this logarithmic scaling in order to obtain an estimation of loudness that is closer to human auditory perception, as per Fechner's law (Dehaene, 2003).

**Figure 3:**
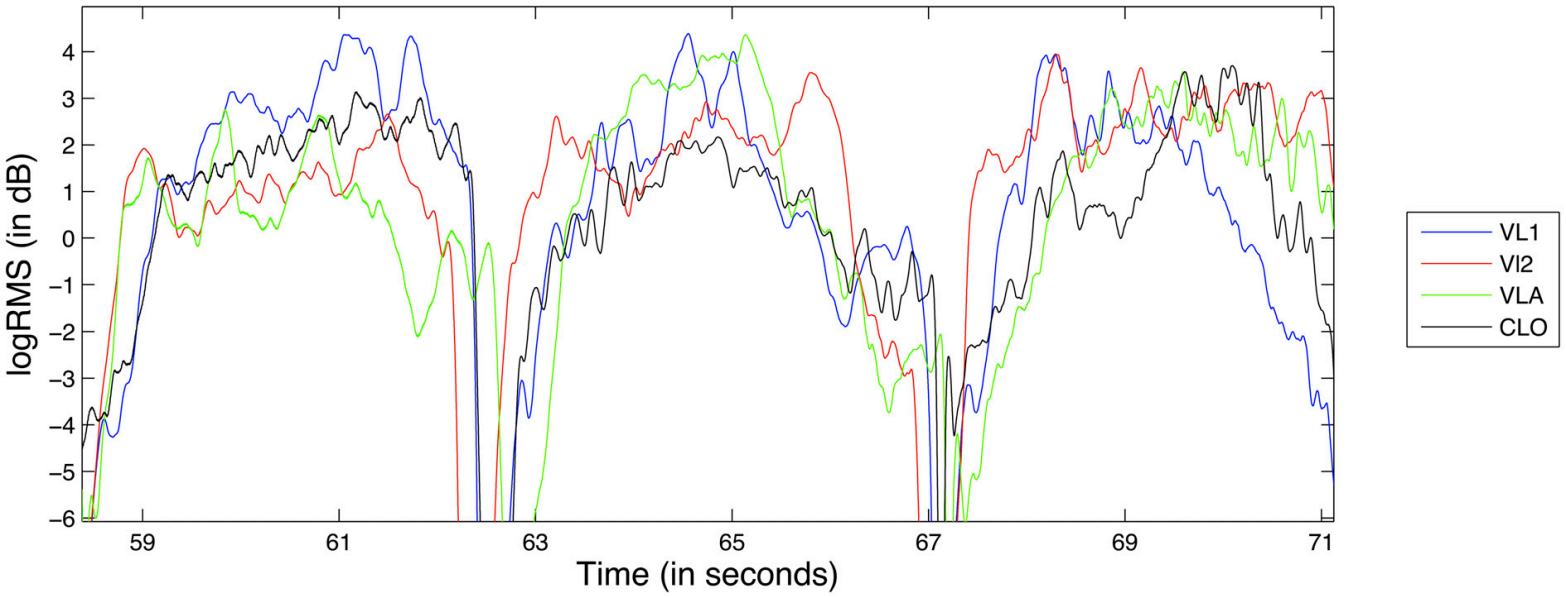
**Excerpt from the logRMS feature from exercise D1, for all instruments**.

Typically, musical scores include both instantaneous as well as gradual changes in dynamics. When the scores of more than one performer feature the same dynamics change at the same time, this can give the impression of coordinated action regardless of whether such coordination actually exists. In order to remove such a bias from our data, and in accordance to previous studies (Papiotis et al., 2012), we subtract a linear trend from the logRMS feature in each individual note in the recording. This way, the note-to-note changes in dynamics are greatly reduced, making temporal fluctuations of dynamics within each note's boundaries much more prevalent. An example of the detrended logRMS feature for the D1 exercise can be seen in Figure 3.

*Timbre* refers to the quality of the sound, and is an elusive concept whose perceptual and quantitative attributes have been the subject of study from many academic viewpoints (Krumhansl, 1989; Hajda et al., 1997). In contrast to the rest of our studied dimensions, timbre cannot be easily studied individually as it is inextricably connected to both dynamics and pitch (Krumhansl and Iverson, 1992). In our analysis, we opted to quantify the timbre characteristics of the performance using two different types of data: instrumental (sound-producing) gesture data as well as audio data.

**Figure 4:**
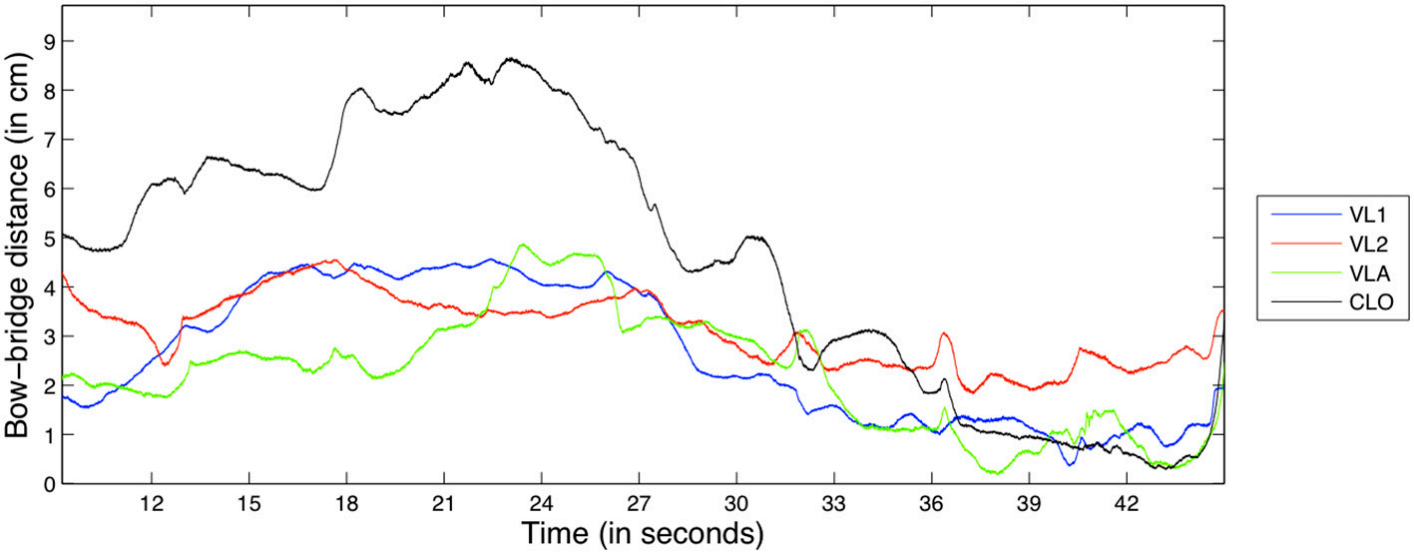
**Excerpt from the bow-bridge distance feature from exercise D1, for all instruments**.

Our gesture-based timbral feature is the *bow-bridge distance*, the distance (in centimeters) from the bridge to the point of contact between the bow and the string that is being excited. Bow-bridge distance is a parameter that is instrumental in the achievement of different timbral qualities in bowed string performance (Schelleng, 1973). Composers and performers use the *sul tasto* or *sul ponticello* (on the bridge and on the fingerboard, respectively) terms to describe bow-bridge distance in written music and manipulate the quality of the produced sound by changing the balance between the amplitude of the fundamental frequency and the higher harmonics. In fact, bow-bridge distance manipulation was also the main focus of the timbre exercise we used in our experiments. An example of the bow-bridge distance feature for the D1 exercise can be seen in Figure 4.

Audio-based timbral features are typically extracted from the audio spectrum: examples of the most prevalent features include the spectral centroid and the Mel-Frequency Cepstral coefficients (MFCCs). As mentioned above, separation between timbre and other dimensions of the performance (dynamics and pitch) is difficult to achieve in a spectral representation. Recent work by Peeters et al. (2011) investigated the redundancy among spectral features using correlational analysis and hierarchical clustering. The authors were able to distinguish ten classes of features that were largely independent from a statistical point of view. Of the available features based on their feature extraction toolbox, we chose to use the *Spectral Crest* feature: a descriptor of the noisiness/harmonicity of the sound that was shown to be relatively independent from other dynamics- or pitch-related features.

Spectral Crest is calculated as follows: first, the audio signal is divided into frames of 23 milliseconds with a 75% overlap. For the *i*_th_ frame of the audio signal, the power spectrum is obtained using the Short-time Fourier Transform, yielding *k* frequency amplitude values *a*_k_ (where *K* is the number of STFT bins). Finally, the Spectral Crest is obtained by comparing the maximum value and arithmetical mean of the power spectrum:

(4) $$SpectralCrest\left(i\right)=\frac{\text{max }a_{k}(i)}{\frac{1}{K}\sum_{k\;=\;1}^{K}a_{k}(i)}$$

**Figure 5:**
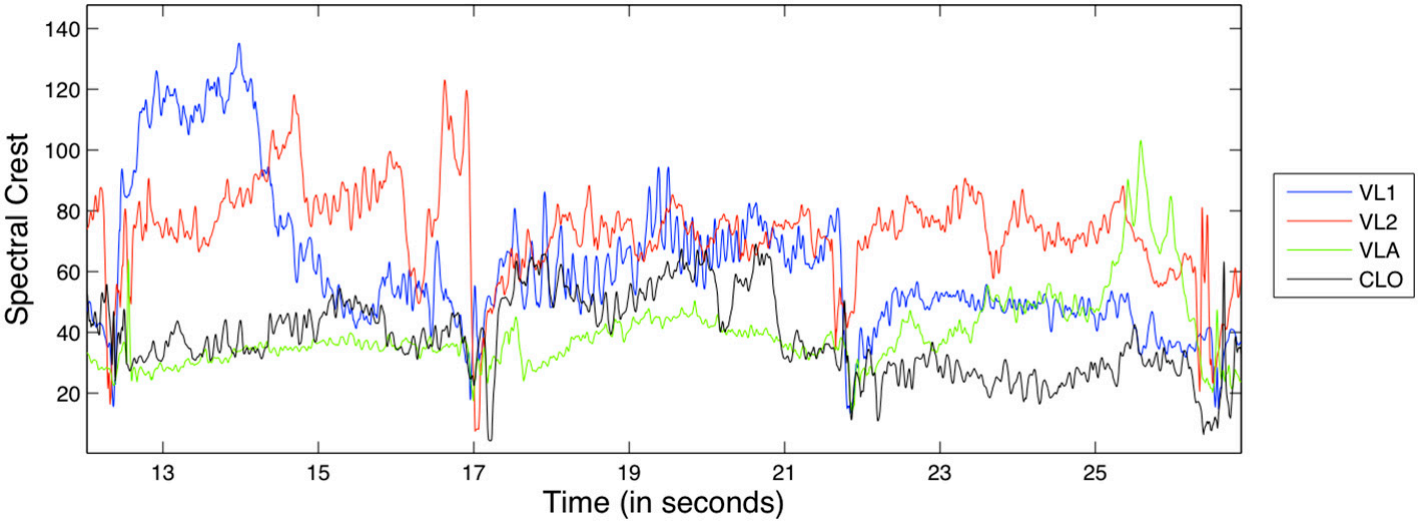
**Excerpt from the spectral crest feature from exercise D1, for all instruments**.

An example of the Spectral Crest feature for the D1 exercise can be seen in Figure 5; as a ratio, it is dimensionless and therefore has no unit.

**Figure 6:**
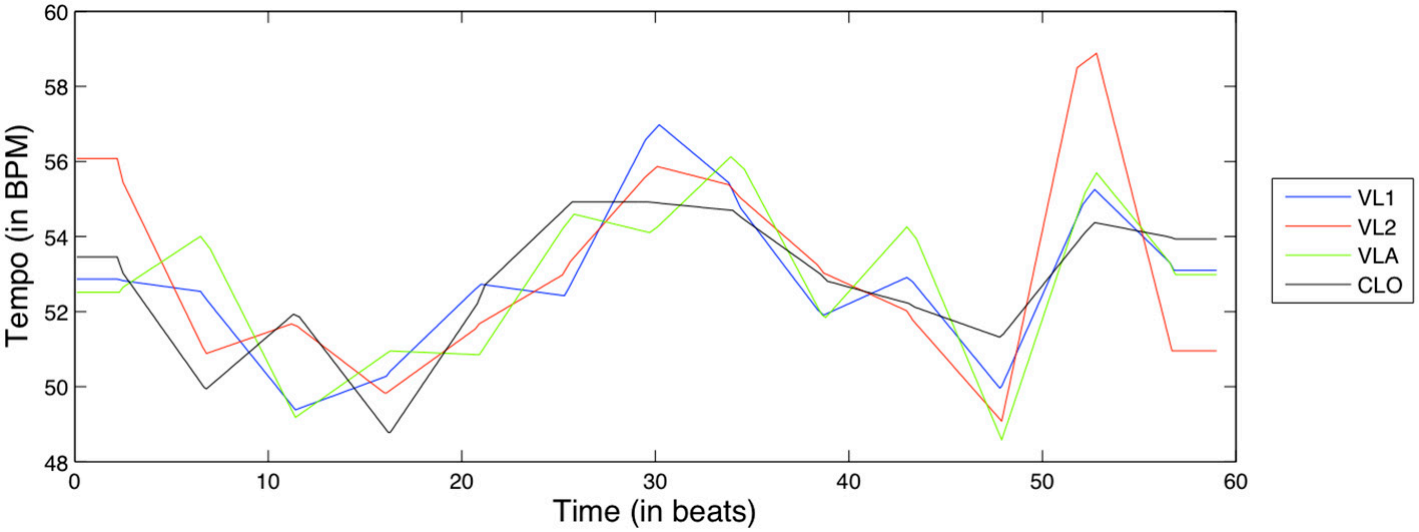
**Tempo curve feature for exercise D1, for all instruments**.

*Tempo* refers to the speed or pace at which a musical composition is performed. In the context of music information retrieval, tempo is typically represented with so-called *tempo curves*, measured in beats-per-minute (BPM). For the *i*_th_ note in a recording, the tempo curve is calculated as follows:

(5) $$Tempo\left(i\right)=\frac{performed\;note\;duration}{score\;note\;duration}*scoreBPM$$

where the performed note duration and score note durations are expressed in seconds and the score *BPM* is the initial *BPM* tempo at which the musicians were instructed to play. After the initial computation, the tempo curve is smoothed at the bar level using a moving average to derive an overall tempo behavior; this way we aim to focus on long-term tempo fluctuations (similar to the period correction mechanism discussed in the introduction) rather than short-term asynchronies. An example of the Tempo curve feature for the D1 exercise can be seen in Figure 6.

The rationale behind using this tempo representation (rather than using e.g., note onset/offset asynchronies) is as follows. First, it was our intention to look at tempo tendencies on a high level of abstraction (as opposed to low-level asynchronicities), and such performance intentions are much more evident on a broader time scale. Second, a time-series representation of tempo allows us to approach it in the same way as for the other performance dimensions when quantifying interdependence.

#### Temporal alignment of solo features

Since in the solo case the musicians were not performing simultaneously, the recordings are temporally mismatched; even if they are manually shifted so that the first note onsets coincide, given time they will start to drift apart up to the point where the same sample index corresponds to different points in the score. In the case of Intonation, Dynamics and Timbre, our objective is to study the musicians' interdependence excluding all other facets of the performance, and it was therefore necessary to time-warp the solo performances in order to compare the features while excluding timing information.

**Figure 7:**
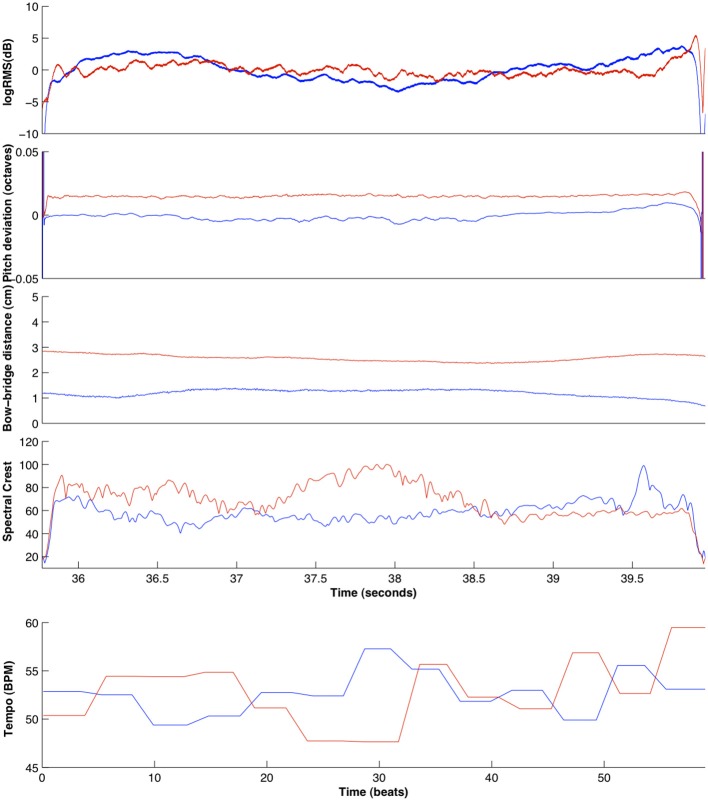
**Comparison of all extracted features for the *ensemble* (blue) and *solo* (red) recordings of violin 1, for an excerpt of exercise D1 (for the pitch deviation, logRMS, bow-bridge distance, and spectral crest features) and the entire exercise (for the tempo curve feature)**.

This was achieved by applying a note-by-note temporal warping algorithm based on resampling the extracted time series (pitch deviation, logRMS, bow-bridge distance, Spectral Crest) between note onsets. For each note in the score, the samples between the note onset and the note offset of the *solo* note are resampled to match the duration of the equivalent *ensemble* note. Then, this segment is temporally shifted so that the temporal position of the note onset and offset matches that of the corresponding note of the *ensemble* recording. All solo recordings were thusly processed to match the ensemble recordings, in order to mimic the natural timing of a joint performance. An example comparison between *ensemble* and *solo* features for violinist 1 in the D1 exercise can be seen in Figure 7.

## Interdependence methods

Having extracted our time series features for each performer and each recording, the next step is to search for interdependence between the features of each member of the quartet. Four methods for measuring interdependence have been tested and will be briefly presented in the following section; for an excellent literature review on these methods we redirect the reader to the one carried out by Pereda et al. (2005). Although the methods vary in terms of complexity as well as the research discipline in which they originate from, we opted to divide the methods based on whether they are most suited for dependences of a linear or nonlinear nature. For each category, there is one symmetric and one directional method; the difference between the two being that a directional method can also assess the direction of influence between two interacting time series besides the strength of the interdependence. Besides introducing the four methods, we also provide some additional details on the way they are computed as well as the parameters that must be selected for computation.

### Linear methods

#### Pearson product-moment correlation coefficient

This is the most common method utilized for quantifying pairwise linear dependence; its output *p_x,y_* between time series *x* and *y* ranges from *p_x,y_* = −1, i.e., complete linear inverse correlation between timeseries *x* and *y*, to *p_x,y_* = 1, i.e., complete linear direct correlation between time series *x* and *y*, with a value *p_x,y_* = 0, suggesting an absence of linear dependence between the two time series. Since linear correlation is calculated pairwise and is symmetric, we calculated the correlation coefficient for each one of the 6 possible pairs between the four musicians. The final correlation values were normalized using the Fisher z-transformation.

It must be noted that indiscriminately applying Pearson Correlation on continuous data regardless of their characteristics (such as autocorrelation or distribution) has been criticized and alternatives such as first-order differencing have been proposed (Schubert, 2002; Upham, 2012). The reason we have opted not to perform such transformations on our time series is two-fold. First, first-order differencing time series that describe performance dimensions such as dynamics or intonation would severely alter their structure, potentially removing critical characteristics of the time series (such as within-note temporal fluctuations) which we wish to maintain. Second, one of the goals of this study is to compare across different methods in terms of their capacity to detect interdependence from the same performance data; if another measure can correctly quantify interdependence without the need for first-order differencing, it should be preferred over a method which requires such transformations.

***Granger causality***. In studying the relationship between time series, it is often useful to assess the directionality of that relationship; besides the overall degree of interdependence, it is also important to draw conclusions about the direction of influence, i.e., whether variable 1 is influencing variable 2 more than the opposite. A method that is capable of giving such an estimate is Granger causality (Granger, 1969), a statistical concept that was first applied to econometrics, and recently to computational neuroscience (Brovelli et al., 2004; Londei et al., 2007). It poses the hypothesis that if time series *x* causes time series *y*, then past values of *x* should significantly help in predicting future values of *y* as opposed to simply using past values of *y* to predict its own future; this is assessed through the use of a multivariate vector autoregressive modeling (or linear regression analysis, depending on the approach). In this study, we used a freely available MATLAB toolbox by Seth (2010).

Since Granger causality is a directional measure, the connectivity analysis of four musicians yields 12 pairwise causality assessments *G_x, y_* (three causal pairs for each variable) as well as the total causal density *G_density_* of the ensemble, a bounded value between 0 and 1, with 0 signifying a complete lack of causal interactivity; we use the latter value as an estimation of the total amount of causal interactivity sustained by the network of musicians.

#### Nonlinear methods

***Mutual information***. Mutual Information is a non-directional measure originating from the field of Information theory. It is not a nonlinear method *per se*, being based on the concept of entropy as proposed by Shannon in the 1950s and therefore dealing with reduction of information rather than the linearity of the data. For a pair of time series *x* and *y*, mutual information measures the difference between two types of Joint Shannon Entropy; the joint entropy of the two variables as measured from the data, and the joint entropy of the two variables as if they were independent (Moddemeijer, 1989; Cover and Thomas, 2006). If they are indeed independent, *MI_x, y_* is zero. Otherwise, *MI_x, y_* is a positive, non-bounded value measured in nats that represents the reduction of uncertainty about *x* that is gained by knowing the outcome of *y* and vice versa. Similar to the case of linear correlation, the analysis of a network of four musicians yields 6 pairwise values of Mutual Information.

***Nonlinear coupling coefficient***. There exists a variety of nonlinear interdependence measures that quantify the signature of directional couplings between two time series *x* and i (Lehnertz, 2011); it is assumed that the processes behind the time series are characterized by separate deterministic dynamics which both exhibit an independent self-sustained motion. Assuming the existence of directionality, i.e., the dynamics of one process driving the dynamics of the other, directional couplings can in principle be detected by quantifying the probability with which close states of the driven dynamics are mapped to close states of the driving dynamics.

The state space of *x* (i.e., the set of all its possible states with each state corresponding to a unique point in the space of that set) is reconstructed using the method of state space embedding; then, a number of spatially nearest neighbors are selected for each point *x_n_* in the state space (excluding temporal neighbors through the use of a given threshold). Finally, the squared mean Euclidean distance from the nearest *k* neighbors of *x_n_* is calculated, along with the *y*-conditioned squared mean Euclidean distance (by replacing the nearest neighbors of *x_n_* by the equal time partners of the closest neighbors of *x_n_*). It has been shown that, when *x* → *y* coupling occurs, there is increased probability that close states of *y* are mapped to close states of *x*. Several available measures based on the above paradigm exist; of these we use the measure *L*, which was recently shown to be of higher sensitivity and specificity for directional couplings than previous approaches (Chicharro and Andrzejak, 2009). The output of *L_x, y_* is a bounded value between 0 and 1, with 0 signifying a complete lack of interdependence. For a more in-depth explanation of this particular method as well as a proper mathematical formulation, we direct the reader to the article where the method was originally introduced (Chicharro and Andrzejak, 2009). As in the case for Granger causality, 12 pairwise calculations of coupling *L_x, y_* are carried out. However, we currently have no method of measuring the overall interdependence of the string quartet; the average value of the set of 12 coupling values is used as estimation instead.

### Details on interdependence estimation and parameter selection

For the *Intonation, Dynamics*, and *Timbre* dimensions, we chose to sequentially compute interdependence on the time series by splitting them into consecutive non-overlapping windows. There are three reasons behind this decision: first, as we are studying features that change with time, it is natural that the amount of interdependence will also vary—something which can potentially reveal the role of the musical score in the performance, as the collaborative task that must be jointly carried out by the musicians. Second, by windowing the time series we can reduce the non-stationarity in our data, thus making the interdependence measures more reliable. Finally, we can deal with smaller amounts of data at a time, which removes the need to downsample our signals in order to cope with memory requirements. For the *Tempo* dimension, we apply the four interdependence methods on the entire tempo curve features, since they are sampled at much larger time intervals and therefore consist of significantly fewer samples.

For the three performance dimensions where a windowed approach was adopted, the time series features are split into windows whose size is calculated in *beats* according to the score. Beat positions are converted to sample indices in order to split the time series using the score-performance alignment. All of the recorded exercises have a bar length of four beats with the exception of the *Intonation*/I1 exercise which has a bar length of six beats; we chose a universal window size of eight beats as a compromise (in all exercises except I1, 8 beats correspond to two bars, while in I1 they correspond to 1.5 bar). Section “Comparison Across Different Exercises and Parameters” provides some further information on how the window size affects the interdependence estimation.

For each window, interdependence strength is calculated using the four methods as described in the previous section (six pairwise values of Pearson Correlation, six pairwise values of Mutual Information, one value of Causal Density, and twelve pairwise values of Nonlinear Coupling). These values are averaged across performer pairs (except for the causal density value which is not pairwise) to produce a single sequence of interdependence values along the duration of each recording. For the *Tempo* dimension, the estimated interdependence values are simply averaged across all performer pairs since there is no windowing.

Besides the window size, other parameters are necessary to be defined. For the case of Granger causality, the order of the multivariate regression model must be chosen. We used the Bayesian Information Criterion in order to automatically select the model order, as it was estimated from each of the features.

For the calculation of the nonlinear coupling coefficient, there are four parameters that must be given as an input to the algorithm:

- *embedding dimension* (*m*), the number of past values to be included for the state space reconstruction
- *time delay parameter* or *tau* (τ), the time delay in samples between each of the past values used
- number of nearest neighbors (k), and
- *Theiler window* (*W*), the threshold used to exclude temporally close points from being selected as nearest neighbors.

**Figure 8:**
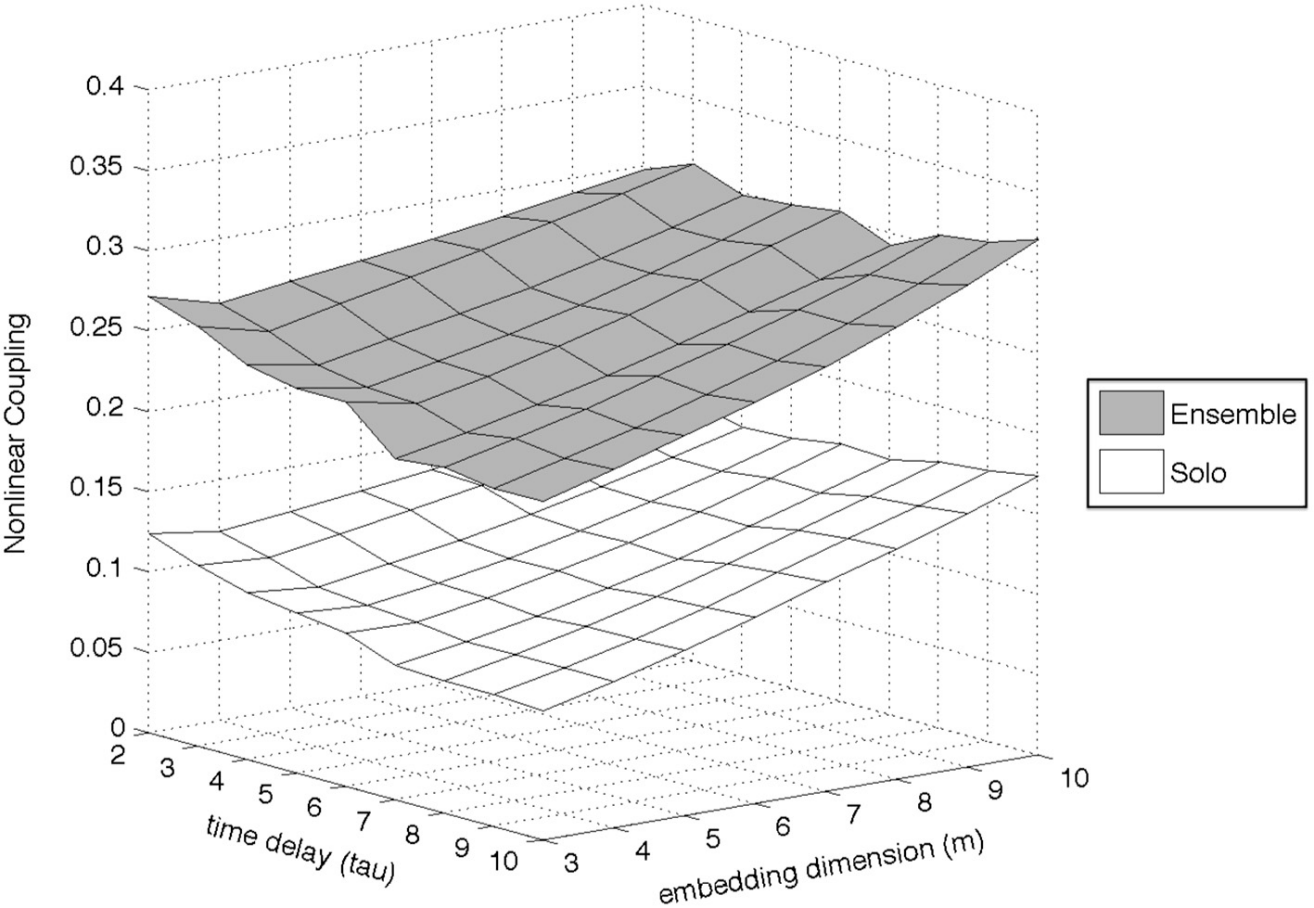
**Example of the effect of parameter choice (embedding dimension and time delay) on the estimation of the Nonlinear Coupling coefficient, for the Dynamics dimension of exercise D1**.

Experimenting with the values of these parameters, it became evident that the most important ones were the embedding dimension (*m*) and the time delay (τ), since they were the ones who had the greatest impact on the outcome of the algorithm; the number of nearest neighbors was set to *k* = 3, and the Theiler window to *W* = 2τ. We experimented with a wide range of values for both *m* and τ (*m* = [3 : 10] and τ = [2 : 10]) and found that the differences between *ensemble* and *solo* recordings were similar irrespective of the *m* and τ values (an example of this observation for the Dynamics dimension of the D1 exercise can be seen in Figure 8). The final parameters used were *m* = 10 and τ = 5.

## Results

In this section, we present the obtained results of our interdependence analysis. First, we report the mean (*μ*) and standard deviation (σ) along with the number of data points (*N*) of interdependence values for each dimension and its corresponding exercise separately. We also report the results of a sequential ANOVA test, in order to decide whether there is a significant difference in mean interdependence strength values between the *ensemble* and *solo* conditions. All ANOVA results reported in this section were additionally confirmed using a non-parametric (Wilcoxon rank-sum) test, which yielded similar results. In the second half of the section, we compare interdependence values across all recorded exercises for each dimension individually over a wide range of window size values (for the Intonation, Dynamics, and Timbre dimensions) as well as smoothing window size values (for the Tempo dimension).

### Intonation

Table 2 contains the averaged interdependence values for each tested method, for the Intonation dimension. The methods were applied on the I1 exercise; we remind the reader that for I1 two separate *ensemble* recordings were carried out (with intonation annotations, and without annotations).

**Table 2:** **Mean (μ) and standard deviation (σ, *N* = 17) of interdependence strength for the Intonation dimension per experimental condition**.

**Method**	**Interdependence strength, intonation (I1)**		
	**Ensemble, with annotations**	**Ensemble, without annotations**	**Solo**
Linear correlation	μ = −0.032	μ = −0.039	μ = 0.001
	σ = 0.564	σ = 0.512	σ = 0.321
Causal density	μ = 0.0322	μ = 0.0317	μ = 0.0082
	σ = 0.0283	σ = 0.0302	σ = 0.0083
Mutual information	μ = 0.464*	μ = 0.415*	μ = 0.277
	σ = 0.211	σ = 0.147	σ = 0.103
Nonlinear coupling coefficient	μ = 0.287*	μ = 0.263*	μ = 0.201
	σ = 0.098	σ = 0.072	σ = 0.068

*Values with an asterisk denote a statistically significant difference between ensemble and solo*.

On initial inspection, it can be observed that the Linear Correlation method is not able to detect any significant differences between the *ensemble* and *solo* recordings of I1. Moreover, marginally negative correlation values between the pitch deviations of the musicians are observed in all three recordings. Given the noisy and nonlinear nature of the pitch deviation time series, this result does not come as a surprise.

Quite the opposite result can be observed for each of the three remaining interdependence methods. All of them estimate a higher amount of interdependence for the two *ensemble* recordings in comparison to the *solo* recording, while the *ensemble-with-annotations* recording demonstrates a slightly higher amount of interdependence in comparison to the *ensemble-without-annotations* recording. Whether this is a result of the order of the recordings, the existence of the annotations or chance, we cannot say; more experimental data are necessary to answer the question.

Although all three methods demonstrate higher interdependence in the *ensemble* conditions in comparison to the *solo* condition, it is difficult to decide from the average interdependence values alone whether that difference is large enough to state that they can indeed capture a significantly higher amount of ensemble coordination in the *ensemble* recordings. In order to assess significance, we ran a repeated measures One-Way ANOVA test on the results of all analysis windows; the results showed that both Mutual Information [*F*_(1, 16)_ = 18.324, *p* = 0.001, η^2^ = 0.71] as well as the Nonlinear Coupling Coefficient [*F*_(1, 16)_ = 16.085, *p* = 0.001, η^2^ = 0.72] could successfully separate the interdependence means of each *ensemble* condition from the *solo* condition. One the other hand, Granger causality did not provide significant separation between *ensemble* and *solo*. None of the interdependence methods found a statistically significant difference between *ensemble-with-annotations* and *ensemble-without-annotations*.

The fact that the non-linear methods perform better than the linear methods (and especially Granger Causality) can be potentially explained by the noisy and non-linear nature of the pitch deviation time series themselves. However, besides these characteristics, there is another important factor that might explain the difference: by subtracting the score-defined pitch (a succession of constant values) from the estimated pitch (a highly autocorrelated time series), we introduce discontinuities that cannot be properly modeled using autoregressive processes, such as the ones employed in the computation of Granger Causality.

It must be also noted that naturally, intonation choices (in terms of how intervals are performed) in the *solo* condition are bound to be quite different from intonation choices in the two *ensemble* conditions, mainly due to the lack of a common goal (i.e., consonant chords) in the *solo* condition. Although not part of our experimental design, an additional *solo* recording that is carried out post-*ensemble* could perhaps shed more light on the effect of such choices on intonation interdependence and vice versa; we return to this topic in the Discussion section of this article.

### Dynamics

Table 3 contains the average interdependence values for the Dynamics dimension. The methods were applied on the D1 and D2 exercises.

**Table 3:** **Mean (μ) and standard deviation (σ, *N* = 7 for exercise D1 and *N* = 6 for exercise D2) of interdependence strength for the Dynamics dimension, per exercise and experimental condition**.

**Method**	**Interdependence strength, dynamics (D1)**		**Interdependence strength, dynamics (D2)**	
	**Ensemble**	**Solo**	**Ensemble**	**Solo**
Linear correlation	μ = 0.343	μ = 0.154	μ = 0.368	μ = 0.279
	σ = 0.375	σ = 0.232	σ = 0.451	σ = 0.349
Causal density	μ = 0.0039	μ = 0.0018	μ = 0.0080	μ = 0.0032
	σ = 0.0043	σ = 0.0014	σ = 0.0019	σ = 0.0012
Mutual information	μ = 0.494*	μ = 0.306	μ = 0.688*	μ = 0.449
	σ = 0.092	σ = 0.067	σ = 0.182	σ = 0.109
Nonlinear coupling coefficient	μ = 0.262*	μ = 0.152	μ = 0.372*	μ = 0.256
	σ = 0.052	σ = 0.042	σ = 0.078	σ = 0.046

*Values with an asterisk denote a statistically significant difference between ensemble and solo*.

The results are quite similar to the Intonation case, save for generally higher amounts of interdependence and slightly different results for the Linear Correlation method. All four methods now seem to detect higher amounts of interdependence for the *ensemble* case, for both recorded exercises. The repeated measures ANOVA test once again showed significant separation between *ensemble* and *solo* only for the Mutual Information [D1: *F*_(1, 6)_ = 21.022, *p* = 0.004, η^2^ = 0.55, D2: *F*_(1, 5)_ = 39.082, *p* = 0.002, η^2^ = 0.90] and the Nonlinear Coupling Coefficient [D1: *F*_(1, 6)_ = 28.048, *p* = 0.002, η^2^ = 0.66, D2: *F*_(1, 5)_ = 28.409, *p* = 0.003, η^2^ = 0.82]. Given how the logRMS signals are detrended in a note-by-note fashion, the same type of discontinuity is present in this case, making the computation of Granger Causality difficult once again.

### Timbre

Table 4 contains the average interdependence values for the Timbre dimension. The methods were applied on the T1 exercise; we remind the reader that for the Timbre performance dimension, two different features are used (bow-bridge distance and spectral crest).

**Table 4:** **Mean (μ) and standard deviation (σ, *N* = 7) of interdependence strength for the Timbre dimension, per feature and experimental condition**.

**Method**	**Interdependence strength, timbre (T1) Bow-bridge distance**		**Interdependence strength, timbre (T1) Spectral crest**	
	**Ensemble**	**Solo**	**Ensemble**	**Solo**
Linear correlation	μ = 0.642	μ = 0.676	μ = 0.158	μ = 0.035
	σ = 0.886	σ = 0.822	σ = 0.397	σ = 0.182
Causal density	μ = 0.0134*	μ = 0.0093	μ = 0.0080	μ = 0.0019
	σ = 0.0048	σ = 0.0040	σ = 0.0102	σ = 0.0018
Mutual information	μ = 1.057	μ = 1.070	μ = 0.341*	μ = 0.170
	σ = 0.128	σ = 0.247	σ = 0.138	σ = 0.033
Nonlinear coupling coefficient	μ = 0.633	μ = 0.617	μ = 0.231*	μ = 0.154
	σ = 0.062	σ = 0.085	σ = 0.076	σ = 0.056

*Values with an asterisk denote a statistically significant difference between ensemble and solo*.

For the case of bow-bridge distance, the results are different from the two previous cases. Neither Linear Correlation nor Mutual Information show higher amounts of interdependence for the *ensemble* condition (although it can be argued that the differences between *ensemble* and *solo* are quite small compared to the previous cases). Causal Density and Nonlinear Coupling show higher values for the *ensemble* condition, although the repeated measures ANOVA test confirms a statistically significant difference only for the Granger Causality measure [*F*_(1, 6)_ = 7.628, *p* = 0.036, η^2^ = 0.76]. Given how the artificially introduced discontinuities do not exist in the bow-bridge distance time series, the relatively good performance of the Granger Causality method (compared to other performance dimensions) is reasonable; after all, Granger Causality has been previously used with success on similar gesture data from string instrument performances (D'Ausilio et al., 2012).

The results on the Spectral Crest feature are a return to the pattern observed in previous results, especially Dynamics. All four methods show higher interdependence values for the *ensemble* condition, with statistical significance for the Mutual Information [*F*_(1, 6)_ = 13.608, *p* = 0.01, η^2^ = 0.63] and Nonlinear Coupling Coefficient methods [*F*_(1, 6)_ = 6.747, *p* = 0.041, η^2^ = 0.66]. Although there are no artificially introduced discontinuities in the Spectral Crest feature, the signal is quite nonstationary—especially around note change events.

### Tempo

Finally, Table 5 contains the average interdependence values for the Tempo dimension. The methods were applied on the R1 and R2 exercises.

**Table 5:** **Interdependence strength for the Tempo dimension, per exercise and experimental condition**.

**Method**	**Interdependence strength, Tempo (R1)**		**Interdependence strength, Tempo (R2)**	
	**Ensemble**	**Solo**	**Ensemble**	**Solo**
Linear correlation	0.983	0.718	0.830	0.523
Causal density	0.697	0.362	0.462	0.160
Mutual information	1.207	0.534	0.715	0.292
Nonlinear coupling coefficient	0.112	0.066	0.063	0.026

All four methods show higher amounts of tempo interdependence for both exercises, with the amount of interdependence in the *ensemble* recordings being more than double the amount of *solo* interdependence (with the exception of Linear Correlation). Unfortunately, the lack of a windowed analysis means that a test for statistical significance for each interdependence method cannot be carried out in this case.

### Comparison across different exercises and parameters

Up to this point, we have only discussed interdependence results in an *ensemble* vs. *solo* context—i.e., without drawing any comparisons across the different exercises for the same performance dimension. Such a comparison would indeed be valuable in assessing the interdependence methods' capacity for not only detecting interdependence, but also quantifying its relative amount.

However, each exercise features a different score—each one containing different numbers of notes, note durations, and types of relations between the musicians' parts. For that reason, the interdependence estimates of different *ensemble* recordings are not directly comparable, as the overall amount of interdependence that is estimated for each piece may vary regardless of the actual amount of cooperation that exists among the performers.

For this reason, we make use of the interdependence estimated from the *solo* recordings as a baseline to which the *ensemble* interdependence can be compared. More specifically, we subtract the estimated *solo* interdependence from the estimated *ensemble* interdependence in order to obtain a relative measure, the amount of “interdependence gain” that is observed as a result of joint action. Given how there is a single *solo* and *ensemble* recording for each exercise, the comparisons reported in this subsection are difficult to generalize or statistically test. Besides the fact that the musicians' actions are not always deliberate or a consequence of interdependence, deviations across repeated performances of the same exercise should be expected. Therefore, the following analysis is intended as an exploration and potential direction for further research rather than a pursuit of definitive conclusions.

In order to draw a fair comparison, we also carry out this analysis over a wide range of values for the window size parameter (for Intonation, Dynamics, and Timbre) and the smoothing window width parameter (for Tempo); this way, we can investigate the effect of these parameters on the estimated interdependence. The Mutual Information method was chosen for the iterative estimation of relative interdependence, due to its rapid computation time as well as its demonstrated capability at quantifying interdependence on our data.

**Figure 9:**
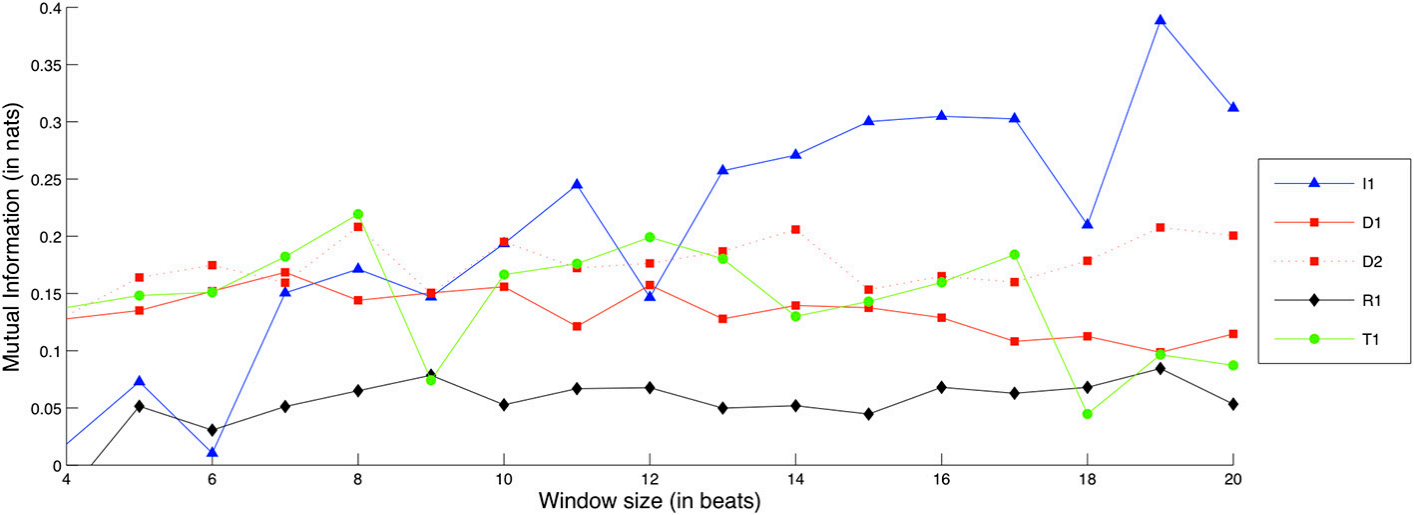
**Interdependence gain for the Intonation dimension, for different exercises and window sizes**. (Legend: I1, Intonation exercise; D1, Dynamics exercise nr.1; D2, Dynamics exercise nr.2; R1, Rhythm exercise nr.1; R2, Rhythm exercise nr.2; T1, Timbre exercise).

Figure 9 shows comparative Mutual Information values for each recorded exercise, in terms of Intonation interdependence.

As it can be observed, the I1 exercise gradually increases in relative interdependence gain as the window size becomes larger, up to the point where it shows notable interdependence gain in comparison to the rest of the exercises. In other words, the larger the temporal context provided, the clearer the importance of Intonation interdependence becomes for the I1 exercise.

Besides I1, the exercises with the next highest interdependence gain appear to be D1, D2, and T1. These exercises along with I1 feature large note durations—a condition which is a reasonable prerequisite for Intonation interdependence. The reader may also observe that the curve of the R2 exercise is absent from the plot: this is because interdependence for the *solo* condition was actually higher than the *ensemble* condition, yielding a curve that dips below zero interdependence gain. As observed in the score excerpt of exercise R2 in Figure 1, the exercise consists of the same two notes for each musician, performed at different degrees of rhythmic syncopation. A possible explanation for this result can be that in the *solo* condition, the performers have no external perturbations in regard to rhythm, and can therefore maintain a much more steady and predictable intonation behavior, which could be mistaken for coordinated action by the interdependence measures. This possibility could be verified or dismissed with the analysis of additional *solo* and *ensemble* recordings of the same exercise, an issue that was commented on previously in this subsection.

**Figure 10:**
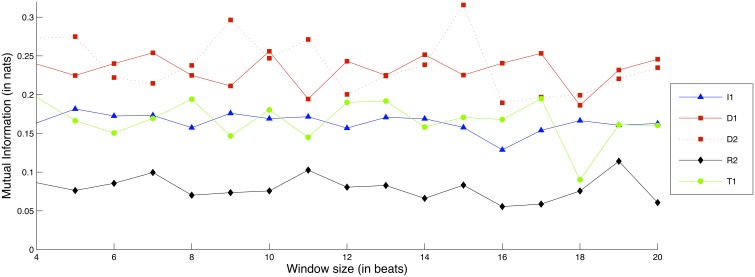
**Interdependence gain for the Dynamics dimension, for different exercises and window sizes**. (Legend: I1, Intonation exercise, D1, Dynamics exercise nr.1, D2, Dynamics exercise nr.2; R1, Rhythm exercise nr.1; R2, Rhythm exercise nr.2; T1, Timbre exercise).

Figure 10 shows comparative Mutual Information values for each recorded exercise, in terms of Dynamics interdependence.

The D1 and D2 exercises consistently demonstrate the highest amounts of interdependence gain, followed closely by the T1 and I1 exercises. Moreover, the window size does not appear to greatly affect the gain of interdependence for either of the exercises. In a similar vein to the Intonation case, the two tempo-based exercises (R1 and R2) demonstrate either low or negative interdependence gain, something that suggests that focus on Dynamics and Intonation does not necessarily imply a concurrent focus on Tempo.

**Figure 11:**
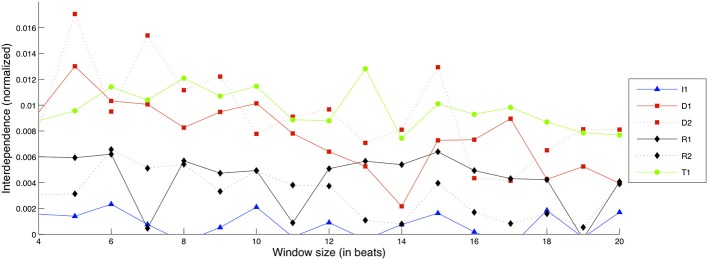
**Interdependence gain for the Timbre dimension, for different exercises and window sizes**. (Legend: I1, Intonation exercise; D1, Dynamics exercise nr.1; D2, Dynamics exercise nr.2; R1, Rhythm exercise nr.1; R2, Rhythm exercise nr.2; T1, Timbre exercise).

For the case of Timbre, rather than using only Mutual Information we decided to combine two results: Causal Density values for the bow-bridge distance feature and Mutual Information for the Spectral Crest feature. The two different estimations were normalized and then added together to create the result seen in Figure 11.

The T1 exercise is shown to sustain a consistent amount of interdependence gain across different frame sizes along with the D1 and D2 exercises, which also show comparable amounts of interdependence gain albeit with larger variation; note here that the scale of interdependence values is much smaller as they are normalized between Mutual Information and Causal Density (which has generally yielded low values for noisy/nonlinear signals, as observed in Tables 2–4). Given how the values reported are a combination of two different analyses (based on bow-bridge distance and Spectral Crest), this could be an indication of “disagreement” between the two sets of results. As mentioned in Section “Materials and Data Processing,” Timbre is a quality of the performance that is difficult to quantify, and the current results show that there is certainly room for improvement in our approach.

**Figure 12:**
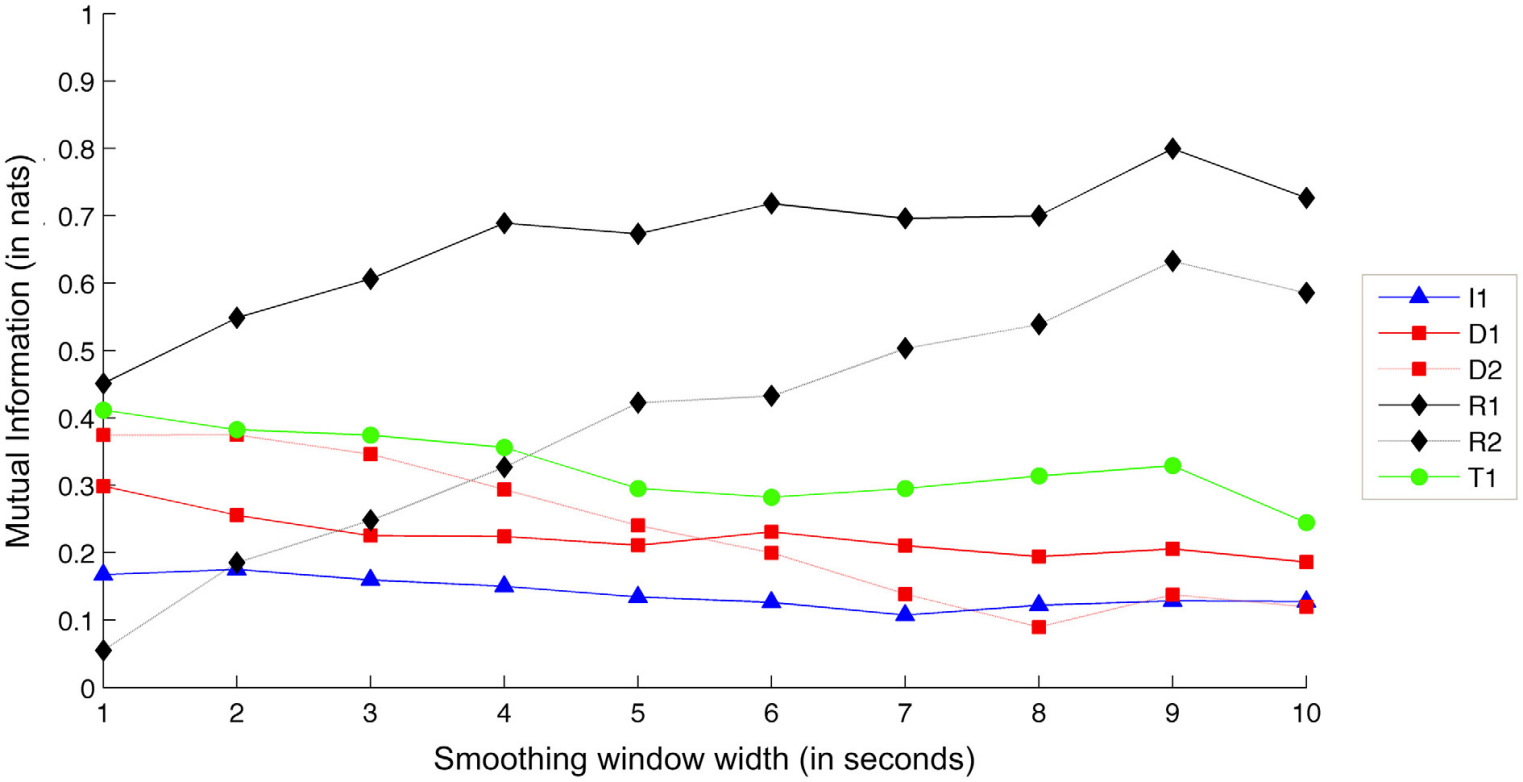
**Interdependence gain for the Tempo dimension, for different exercises and smoothing window sizes**. (Legend: I1, Intonation exercise; D1, Dynamics exercise nr.1; D2, Dynamics exercise nr.2; R1, Rhythm exercise nr.1; R2, Rhythm exercise nr.2; T1, Timbre exercise).

Finally, Figure 12 shows comparative Mutual Information values for each recorded exercise, in terms of Tempo interdependence.

As expected, the two tempo-based exercises (R1 and R2) demonstrate the largest amounts of interdependence gain. Increasing the smoothing window appears to lead to an increase in interdependence gain for the two exercises—a result that, as in the case of Intonation, suggests that larger context leads to clearer view at the underlying goal of the exercise. In support of this, the rest of the exercises maintain a steady level of interdependence gain.

## Discussion

In this article, we carried out a data-driven analysis of interdependence among the members of a performing string quartet. Focusing on Intonation, Dynamics, Timbre, and Tempo as distinct dimensions of the performance, we evaluated a set of measures capable of quantifying musical interdependence in terms of these dimensions, and designed a Music Information Research (MIR)-oriented methodology for the extraction of performance descriptors on which the interdependence measures were applied.

The obtained results suggest that, at least for the simple exercises that were studied, it is feasible to correctly discriminate between recordings where no interaction between the performers exists, and recordings where *some* interdependence can be safely assumed to exist; and the nonlinear interdependence methods appear to be the most suitable for such an analysis. By measuring the difference in interdependence between the *solo* and *ensemble* recordings, the results suggest that it is also feasible to measure how much the performers are interacting in one performance dimension across different exercises and relate the results to the shared goal of the ensemble as it is defined by the underlying score.

We view the main contribution of this work as the inclusion of the different interdependence measures and the evaluation of their potential to capture musical interdependence in several distinct dimensions of the performance. Although the results we have obtained so far are promising, they serve an exploratory rather than conclusive purpose: while a clear tendency is observed (statistically significant interdependence differences between *ensemble* and *solo* for the “target” performance dimension in all exercises), the potential for decisive conclusions is limited by the amount of data that we have collected (in terms of repetitions for each experimental condition). This is especially relevant in the case of Section “Comparison Across Different Exercises and Parameters,” where the *solo* recordings are used as a reference interdependence level to which the *ensemble* recordings are compared. While the results hint at how interdependence is a quantity that varies with the goal of the performed exercise, more repetitions for the two experimental conditions would be necessary in order to distinguish findings that are due to deliberate action rather than “noise” resulting from the innate variability of music performance, a persistent challenge of academic work on the subject (Palmer, 1997).

In the introduction, we discussed how performance dimensions such as Intonation, Dynamics, and Timbre are quite underrepresented in the current literature. The results we have obtained show that it is feasible to include such parameters in future studies, and their inclusion could expand and consolidate aspects of our approach for the cases where our data were not enough to offer strong conclusions. Regarding tempo and/or timing, most available works deal with ensemble coordination from a low-level synchronization point of view (Repp, 2001; Repp and Keller, 2010) and are based on the thoroughly studied *phase correction* and *period correction* phenomena and their mathematical background. A joint analysis of temporal behavior data using both this approach as well as the high-level approach that is followed in our study could highlight their differences or common ground and provide broader description of joint musical action in terms of temporal coordination. Similarly, the inclusion of these methods in the work presented here could help in bridging the conceptual gap between the concepts of synchronization and interdependence.

The materials and experimental conditions considered in this study cover but a small area of the complex phenomenon that is string quartet performance. The experiments were based on a relatively small amount of short exercises, recorded by a single quartet. Longer and more complicated scores could further test the usefulness of our methodology, or reveal interactions between the performance dimensions that were not seen in this study. Studying more quartets with a different potential for coordination could validate or challenge the patterns that we have observed so far, while capturing more repetitions of the same performance along a larger time window could reveal information about how interdependence is established during an ensemble's training process. In our experiment, recordings carried out in the *solo* condition were designed as “ground truth” which was as far removed from ensemble performance as possible; however, it would be also useful to test with more variations of the *solo* condition such as performing alone after having performed together, or even recordings where the musicians are performing a piece together for the first time.

In this study, we represent each performance dimension with numerical features extracted from the recorded data. The features that were selected in our approach are but a subsample of a very large set of performance descriptors that can be found in the Music Information Research literature (Serra et al., 2013); and the proposed methodology could be greatly consolidated by including more features and assessing their capacity to capture musical interdependence.

From our results, we have found that interdependence methods capable of detecting nonlinear interactions are generally more suited for the type of data we tested. One reason could be that the encountered interactions are indeed of a nonlinear nature. Other possible reasons could be the noisy and nonlinear nature of the data, the processing that they undergo in our methodology (such as the introduced discontinuities we discuss in Section Intonation), or a combination of the two. Earlier tests with non-parametric correlation measures (such as Spearman's rank correlation) yielded results similar to the ones obtained by Pearson correlation. The inclusion of more features with different characteristics regarding stationarity, linearity etc. could help in addressing the above questions, and provide more arguments toward the use of these methods in practical applications.

As is usually the case with quantitative research that combines different algorithms and feature extraction techniques, each step requires technical decisions regarding the selection of appropriate parameters, the impact of which on the final result is not always straightforward to predict and often requires iterative computations in order to assess. A clear example of parameter impact can be seen in Section “Comparison Across Different Exercises and Parameters,” where the size of the interdependence analysis window is shown to affect the interdependence difference between the *solo* and *ensemble* cases. Another example of the second point (need for iterative computation over different combinations of parameters) can be seen in Figure 8; although for this analysis we did not discover a notable impact of the Nonlinear Coupling coefficient's parameters on the results, such iterative computations are necessary in order to assess their impact.

In order to assess the interdependence between musicians in one performance dimension vs. another, it is necessary to have reference recordings where (ideally) no interaction between the performers takes place (such as the *solo* recordings in this study). Data collection under these conditions is a complicated and costly process that can only be carried out under experimental conditions. On the contrary, capturing the performance of a quartet under natural conditions is relatively straightforward and commonplace even within the recording industry. To this end, we are currently working to eliminate the need for such reference recordings and making our methodology more easily applicable; our first steps in this direction take advantage of techniques originating from Surrogate Time Series analysis techniques (Schreiber and Schmitz, 2000), and attempt to generate surrogate time series solely from the *ensemble* recordings as a baseline reference.

Two important aspects of musical interdependence that are not addressed in this study are (i) the fluctuation of interdependence strength along time, and (ii) the interpersonal relationships among the musicians and the roles they imply. In its current state, our methodology allows for the investigation of both time-varying estimations (through the use of a windowed analysis) as well as separate estimations for each pair of performers, even though we have not dealt with such questions so far. We aim to advance our research in both of these areas in future studies.

This work has been focused on an ensemble of bowed string instruments. However, it can be reasonably assumed that the presented methodology can be applied to different kinds of ensembles; while ensembles such as wind sections or even singing voice ensembles are an obvious choice, the methodology pertaining to dynamics and tempo can be easily applied to most musical instruments. In fact, the core of our methodology—obtaining numerical representations of the behavior of interacting agents and assessing the interdependence between them—could be useful in studying interactions in social contexts beyond music performance; an example would be the academic discipline of Social Signal Processing (Pantic et al., 2011), where multimodal data originating from human non-verbal behavior are automatically analyzed to study social interaction and derive context-aware causal relationships between interacting agents.

### Conflict of interest statement

The authors declare that the research was conducted in the absence of any commercial or financial relationships that could be construed as a potential conflict of interest.

## References

[B1] Brovelli A. Ding M. Ledberg A. Chen Y. Nakamura R. Bressler S. L. (2004). Beta oscillations in a large-scale sensorimotor cortical network: directional influences revealed by Granger causality. Proc. Natl. Acad. Sci. U.S.A. 101, 9849–9854 10.1073/pnas.0308538101 15210971 PMC470781

[B2] Chicharro D. Andrzejak R. (2009). Reliable detection of directional couplings using rank statistics. Phys. Rev. E 80:026217 10.1103/PhysRevE.80.026217 19792241

[B3] Cover T. Thomas J. (2006). Elements of Information Theory, 2nd Edn Hoboken, NJ: Wiley-Interscience

[B4] D'Ausilio A. Badino L. Li Y. Tokay S. Craighero L. Canto R. (2012). Leadership in orchestra emerges from the causal relationships of movement kinematics. PLoS ONE 7:e35757 10.1371/journal.pone.0035757 22590511 PMC3348913

[B5] Davidson J. W. King E. C. (2004). Strategies for ensemble practice, in Musical Excellence. Strategies and Techniques to Enhance Performance, ed Williamon A. (New York, NY: Oxford University Press), 105–122 10.1093/acprof:oso/9780198525356.003.0006

[B6] De Cheveigné A. Kawahara H. (2002). YIN, a fundamental frequency estimator for speech and music. J. Acoust. Soc. Am. 111, 1917–1930 10.1121/1.1458024 12002874

[B7] Dehaene S. (2003). The neural basis of the Weber–Fechner law: a logarithmic mental number line. Trends Cogn. Sci. 7, 145–147 10.1016/S1364-6613(03)00055-X 12691758

[B8] Devaney J. Ellis D. P. W. (2008). An empirical approach to studying intonation tendencies in polyphonic vocal performances. J. Interdiscipl. Music Stud. 2, 141–156

[B9] Glowinski D. Mancini M. Cowie R. Camurri A. Chiorri C. Doherty C. (2013). The movements made by performers in a skilled quartet: a distinctive pattern, and the function that it serves. Front. Psychol. 4:841 10.3389/fpsyg.2013.00841 24312065 PMC3826428

[B10] Goebl W. Palmer C. (2009). Synchronization of timing and motion among performing musicians. Music Percept. 26, 427–438 10.1525/mp.2009.26.5.427

[B11] Goodman E. (2002). Ensemble performance, in Musical Performance: A Guide To Understanding, ed Rink J. (Cambridge: Cambridge University Press), 153–167

[B12] Granger C. W. J. (1969). Investigating causal relations by econometric models and cross-spectral methods. Econometrica 37, 424–438 10.2307/1912791

[B13] Hajda J. M. Kendall R. A. Carterette E. C. Harshberger M. L. (1997). Methodological issues in timbre research, in The Perception and Cognition of Music, eds Deliege I. Sloboda J. A. (Hove: Psychology Press), 253–306

[B14] Heimann M. (2007). Exercises for string quartet, in European String Teachers Association (Denmark Branch), eds Deckert H. E. Marcus F. (Copenhagen: ACMP Chamber Music Network).

[B15] Kalin G. (2005). Formant Frequency Adjustment in Barbershop Quartet Singing. Stockholm: KTH Royal Institute of Technology

[B16] Keller P. E. (2008). Joint action in music performance, in Enacting Intersubjectivity: a Cognitive and Social Perspective to the Study of Interactions, eds Morganti F. Carassa A. Riva G. (Amsterdam: IOS press), 205–221

[B17] Keller P. E. Appel M. (2010). Individual differences, auditory imagery, and the coordination of body movements and sounds in musical ensembles. Music Percept. 28, 27–46 10.1525/mp.2010.28.1.27

[B18] Krumhansl C. L. (1989). Why is musical timbre so hard to understand, in Structure and Perception of Electroacoustic Sound and Music, eds Nielzen S. Olsson O. (Amsterdam: Elsevier), 43–53

[B19] Krumhansl C. L. Iverson P. (1992). Perceptual interactions between musical pitch and timbre. J. Exp. Psychol. Hum. Percept. Perform. 18, 739 1500873 10.1037//0096-1523.18.3.739

[B20] Lehnertz K. (2011). Assessing directed interactions from neurophysiological signals–an overview. Physiol. Meas. 32, 1715–1724 10.1088/0967-3334/32/11/R01 22027099

[B21] Londei A. D'Ausilio A. Basso D. Sestieri C. Del Gratta C. Romani G. L. (2007). Brain network for passive word listening as evaluated with ICA and Granger causality. Brain Res. Bull. 72, 284–292 10.1016/j.brainresbull.2007.01.008 17452288

[B22] Luck G. Toiviainen P. (2006). Ensemble musicians' synchronization with conductors' gestures: an automated feature-extraction analysis. Music Percept. 24, 189–200 10.1525/mp.2006.24.2.189

[B23] Maestre E. (2009). Modeling Instrumental Gestures: an Analysis/Synthesis Framework for Violin Bowing. Barcelona: Universitat Pompeu Fabra

[B24] Mason J. A. (1960). Comparison of solo and ensemble performances with reference to Pythagorean, just, and equi-tempered intonations. J. Res. Music Educ. 8, 31 10.2307/3344235

[B25] Moddemeijer R. (1989). On estimation of entropy and mutual information of continuous distributions. Signal Process. 16, 233–246 10.1016/0165-1684(89)90132-1

[B26] Moore G. P. Chen J. (2010). Timings and interactions of skilled musicians. Biol. Cybern. 103, 401–414 10.1007/s00422-010-0407-5 21046143

[B27] Nickerson J. F. (1949). Intonation of solo and ensemble performance of the same melody. J. Acoust. Soc. Am. 21, 593 10.1121/1.1906555

[B28] Palmer C. (1997). Music performance. Annu. Rev. Psychol. 48, 115–138 10.1146/annurev.psych.48.1.115 9046557

[B29] Pantic M. Cowie R. D'Errico F. Heylen D. Mehu M. Pelachaud C. (2011). Social signal processing: the research agenda, in Visual Analysis of Humans, eds Moeslund T. B. Hilton A. Krüger V. Sigal L. (London: Springer), 511–538 10.1007/978-0-85729-997-0_26

[B30] Papiotis P. Marchini M. Maestre E. (2012). Computational analysis of solo versus ensemble performance in string quartets: dynamics and intonation, in 12th International Conference of Music Perception and Cognition, eds Cambouropoulos E. Tsougras C. Mavromatis P. Pastiades K. (Thessaloniki).

[B31] Pecenka N. Keller P. E. (2009). Auditory pitch imagery and its relationship to musical synchronization. Ann. N.Y. Acad. Sci. 1169, 282–286 10.1111/j.1749-6632.2009.04785.x 19673794

[B32] Peeters G. Giordano B. L. Susini P. Misdariis N. McAdams S. (2011). The timbre toolbox: extracting audio descriptors from musical signals. J. Acoust. Soc. Am. 130, 2902–2916 10.1121/1.3642604 22087919

[B33] Pereda E. Quiroga R. Q. Bhattacharya J. (2005). Nonlinear multivariate analysis of neurophysiological signals. Prog. Neurobiol. 77, 1–37 10.1016/j.pneurobio.2005.10.003 16289760

[B34] Rasch R. A. (1988). Timing and synchronization in ensemble performance, in Generative Processes in Music: the Psychology of Performance, Improvisation, and Composition, ed Sloboda J. (Oxford: Clarendon Press), 70–90

[B35] Repp B. H. (2001). Processes underlying adaptation to tempo changes in sensorimotor synchronization. Hum. Mov. Sci. 20, 277–312 10.1016/S0167-9457(01)00049-5 11517673

[B36] Repp B. H. (2005). Sensorimotor synchronization: a review of the tapping literature. Psychon. Bull. Rev. 12, 969–992 10.3758/BF03206433 16615317

[B37] Repp B. H. Keller P. E. (2010). Self versus other in piano performance: detectability of timing perturbations depends on personal playing style. Exp. brain Res. 202, 101–110 10.1007/s00221-009-2115-8 20012533

[B38] Schelleng J. C. (1973). The bowed string and the player. J. Acoust. Soc. Am. 53, 26 10.1121/1.1913322

[B39] Schreiber T. Schmitz A. (2000). Surrogate time series. Phys. D Nonlinear Phenom. 142, 346–382 10.1016/S0167-2789(00)00043-9

[B40] Schubert E. (2002). Correlation analysis of continuous emotional response to music: correcting for the effects of serial correlation. Music Sci. 5, 213–236 10.1177/10298649020050S108

[B41] Serra X. Magas M. Benetos E. Chudy M. Dixon S. Flexer A. (2013). Roadmap for Music Information Research. Geoffroy Peeters.

[B42] Seth A. K. (2010). A MATLAB toolbox for Granger causal connectivity analysis. J. Neurosci. Methods 186, 262–273 10.1016/j.jneumeth.2009.11.020 19961876

[B43] Upham F. (2012). Limits on the application of statistical correlations to continuous response data, in Proceedings of the 12th International Conference on Music Perception and Cognition, eds Cambouropoulos E. Tsougras C. Mavromatis P. Pastiades K. (Thessaloniki), 1037–1041

[B44] Wing A. M. Endo S. Bradbury A. Vorberg D. (2014). Optimal feedback correction in string quartet synchronization. J. R. Soc. Interface 11, 20131125 10.1098/rsif.2013.1125 24478285 PMC3928944

[B45] Young V. M. Colman A. M. (1979). Some psychological processes in string quartets. Psychol. Music 7, 12–18 10.1177/030573567971002

